# Inclusion of antimicrobial resistance in a pandemic agreement: why it matters and what comes next?

**DOI:** 10.1093/haschl/qxag044

**Published:** 2026-02-28

**Authors:** Jesic Beckham, Rishabh Jain, Delaram Haghgozar, Enrique Castro-Sanchez, Jyoti Joshi, Mirfin Mpundu, Rifat Atun, Raheelah Ahmad, SEDRIC (Surveillance and Epidemiology of Drug-Resistant Infections Consortium) Board, Dawn Sievert, Direk Limmathurotsakul, Jyoti Joshi, Kamini Walia, Mirfin Mpundu, Nick Feasey, Rifat Atun, Rogier van Doorn, Sharon Peacock, Souha Kanj, Stuart Reid

**Affiliations:** Department of Global, Public and Population Health and Policy, School of Health and Medical Sciences, City St Georges, University of London, London EC1V 0HB, United Kingdom; Department of Global, Public and Population Health and Policy, School of Health and Medical Sciences, City St Georges, University of London, London EC1V 0HB, United Kingdom; Department of Global, Public and Population Health and Policy, School of Health and Medical Sciences, City St Georges, University of London, London EC1V 0HB, United Kingdom; Department of Infectious Disease, School of Medicine, Imperial College London, St Mary's Hospital, London W2 1NY, United Kingdom; International Centre for Antimicrobial Resistance Solutions, Copenhagen 2300 S, Denmark; ReAct Africa, Lusaka, 10101, Zambia; Department of Health Policy and Management, Harvard T.H. Chan School of Public Health, Boston, MA 02115, United States; Department of Global, Public and Population Health and Policy, School of Health and Medical Sciences, City St Georges, University of London, London EC1V 0HB, United Kingdom; Department of Infectious Disease, School of Medicine, Imperial College London, St Mary's Hospital, London W2 1NY, United Kingdom

**Keywords:** pandemic agreement, antimicrobial resistance, pandemic treaty, international health regulations, pandemic instrument, pandemic accord, health equity

## Abstract

**Background:**

Antimicrobial resistance (AMR), referred to as the “constant pandemic,” exceeds malaria and HIV as a cause of mortality across low- and middle-income countries. As AMR has been included in the recently adopted world's first pandemic agreement, we assessed the implications going forward for addressing AMR and meeting the UN General Assembly AMR targets.

**Methods:**

A rapid literature review was conducted to synthesize policy perspectives and empirical literature using 3 databases (PubMed, Embase, and CABI—Global Health) for studies published from December 2021 to May 2025.

**Results:**

Of the 56 included studies, only 2 were empirical research. Inductive and deductive analyses using the Organization for Economic Co-operation and Development framework with a force-field analysis were used to identify drivers and factors that may impede AMR reduction via the pandemic agreement. Challenges include inequity, inadequate governance, and financing. Factors that may impede implementation of the agreement currently outweigh driving forces.

**Conclusion:**

While AMR is included in the pandemic agreement, assessing the merits and risks associated with doing so is important to inform the detail and implementation strategy of the agreement itself. There is consensus that strengthening governance frameworks, fostering equity, and ensuring fair access to health resources are imperative.

Key Takeaways

- Positioning antimicrobial resistance within the pandemic agreement is a positive step, but more work is needed to inform implementation at the national level from a health systems perspective.
- Strengthening governance frameworks, fostering equity, and ensuring fair access to health resources are imperative, and there is consensus on the criticality of these dimensions.
- The lack of empirical data and analysis to substantiate positions highlights the need for monitoring and evaluation going forward.

## Introduction

Antimicrobial resistance (AMR) poses a significant threat globally, responsible for an estimated 1.27 million deaths directly and 4.95 million deaths indirectly in 2021.^1^ The World Bank projected that, by 2050, AMR could reduce global GDP (Gross Domestic Product) by 3.8%, with low- and middle-income countries (LMICs) being most vulnerable. Antimicrobial resistance also drives significant health care expenditures—up to 25% in low-income countries, 15% in middle-income countries, and 6% in high-income countries (HICs).^2^ International efforts to address AMR have gained significant momentum, reflected by the high-level commitments made during the 79th United Nations General Assembly (UNGA) 2024,^3^ where global leaders agreed on a political declaration aiming to reduce the 4.95 million deaths associated with bacterial AMR by 10% by 2030.^3^ The declaration makes 62 recommendations and 45 commitments, including commitment to sustainable national financing, and allocates US$100 million to support at least 60% of countries in developing and funding National Action Plans (NAPs) on AMR by 2030.^4^ As with pandemics caused by other micro-organisms, AMR extends across all forms of biodiversity, affecting plants, animals, and humans. Effective management of AMR requires multisectoral collaboration and is reflected in the monitoring and evaluation of the global action plan (2019) on AMR,^5,6^ developed and supported by the Quadripartite alliance of key United Nations (UN) organizations, including the World Health Organization (WHO), the World Organization for Animal Health (WOAH), the Food and Agriculture Organization (FAO), and the UN Environment Program (UNEP).^7^ A comparative analysis of national responses to the SARS-CoV-2 (COVID-19) pandemic revealed that the most successful strategies involved effective multisectoral coordination directly reporting to the highest levels of government.^8,9^ The COVID-19 pandemic revealed critical gaps in global health security,^10^ such as limitations to the International Health Regulations (IHR) reporting system and lack of broader will, including to commit resources that could improve core capacities following the IHR.

### Policy timeline

To recap, the existing IHR^11,12^ instrument of international law is legally binding for 194 WHO member states. This binding agreement requires countries to improve their core capacities, including legislation, coordination, and surveillance, to detect and respond to national health emergencies. Declaring public health emergencies of international concern (PHEIC) is a cornerstone of the IHR.^10^ The IHR primarily address capacities at a national level, which does not necessarily improve global oversight and coordination.^13,14^ This prompted the WHO to propose a pandemic convention, agreement, or international instrument and initiate revision to the existing IHR. This proposal aimed to make countries better prepared and protected in order to prevent and respond to future pandemics.^15^ First introduced at the March 2021 World Health Assembly (WHA), the proposed convention, agreement, or other international instrument on pandemic prevention, preparedness, and response was meant to enhance global pandemic preparedness and coordination while being underpinned by the IHR.^15^ To support these efforts, the WHO established the Intergovernmental Negotiating Body (INB) in December 2021,with subsequent release of a conceptual draft for the WHO Convention Agreement (referred to as the Zero Draft)^16,17^ and a revised version in April 2024.^18^ During the 12th meeting of the INB (December 2024), progress in research and development (Article 9), local production (Article 10), and regulatory system strengthening (Article 14) was reported, with plans to finalize and implement the pandemic agreement by the 2025 WHA.^19,20^ After 3 years of negotiations, the pandemic agreement was finalized in May 2025, outlining a framework for strengthening international collaboration, equity, and resilience, while several key annexes such as the Pathogen Access and Benefit-Sharing (PABS) mechanism are still under negotiation and implementation planning.^21^ In parallel, amendments to the IHR adopted in May 2024 focused on strengthening national capacities, enhancing multisectoral coordination, improving access to medical products, and securing sustainable financing to address systemic weaknesses in global health governance.^22^

Despite some agreement on the need for revisions to existing mechanisms, disagreements among member states persisted over key provisions and acceptability of an agreement on issues surrounding research and development, technology transfer, access and benefit-sharing, and international cooperation.^23^ The global effort to mitigate acute pandemics and AMR is challenged by limited resources and suboptimal infrastructure.^24^ Low- and middle-income countries are disproportionately affected due to gaps in stewardship, awareness, surveillance, availability of and access to quality care, diagnostics, effective vaccines and drugs, and health care expenditures as well as underperforming health systems.^25^ Less than 50% of countries meet the global Universal Health Coverage index score.^26^ The current draft of the finalized pandemic agreement has a limited focus on AMR, which pertains to pandemic prevention and surveillance in Article 4.^27^ Similarly, regulations specific to AMR are absent from the current IHR, which is potentially a missed opportunity to address this critical global health threat through legally binding agreements.^11,12^

While AMR is described as the “constant,” “silent,” or “slow burning” pandemic, there has been much debate on the merits and potential risks of its inclusion in a pandemic agreement. With the recent adoption of the pandemic agreement, which does include AMR, it is important to continue to gather insights to the consequent benefits and further work needed to accelerate progress in addressing AMR. We conducted this rapid literature review aiming to address the following overarching research question: With the urgent need to address AMR, what are the implications of including or excluding AMR in the pandemic agreement? This question is answered in the context of the wider barriers and drivers for implementation of an agreement. This review seeks to provide timely insights for policymakers and stakeholders so that the detailed articulation and implementation of the agreement can be informed by this synthesis.

## Methods

**Figure 1.**
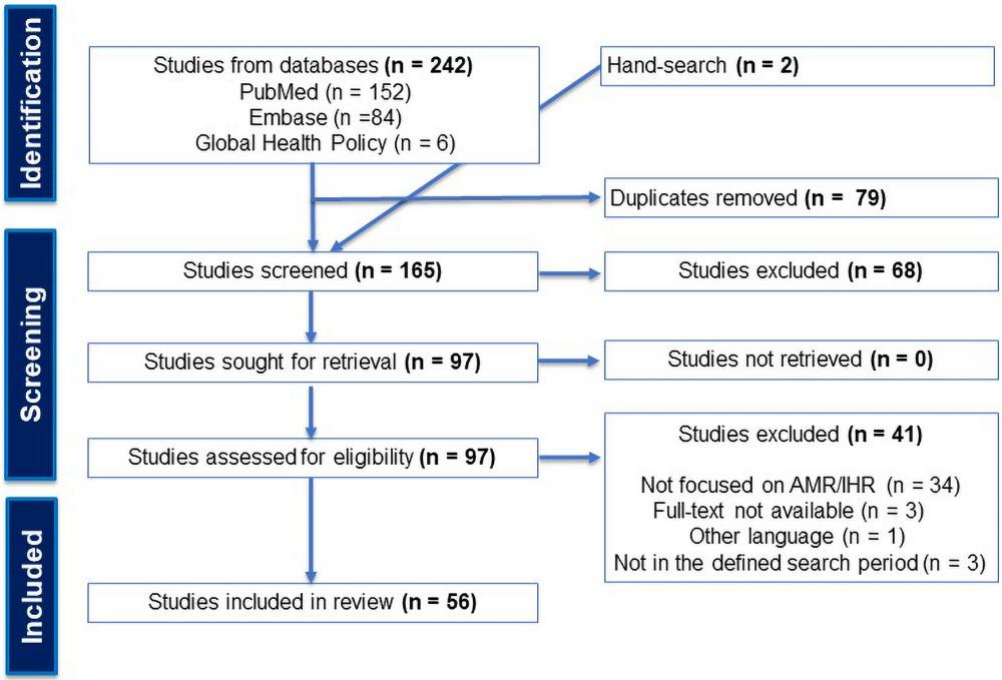
PRISMA flow diagram. Abbreviations: AMR, antimicrobial resistance; IHR, International Health Regulations; PRISMA, Preferred Reporting Items for Systematic reviews and Meta-Analyses.

Our initial scope of the literature revealed few studies based on primary empirical data and hence a systematic literature review was ruled out. A rapid literature review was deemed the most feasible option for achieving timely insights while maintaining a rigorous quality standard.^28^ This process followed the 4-stage Preferred Reporting Items for Systematic reviews and Meta-Analyses (PRISMA) framework: identification, screening, eligibility, and final inclusion^29^ (Figure 1).

### Search strategy and selection criteria

We searched 3 databases (PubMed, Embase, and CABI—Global Health) for articles published between December 1, 2021, and May 31, 2025. This time frame was chosen to align with the special session of the WHA in December 2021, where the proposal was first introduced. Our search was aimed at identifying articles discussing the inclusion of either AMR or the IHR within the context of the pandemic agreement. We used consistent search terms across all databases (the complete list is available in the Supplementary File 01), including the following: “pandemic treaty,” “pandemic accord,” “pandemic instrument,” “pandemic agreement,” AND [(“AMR” or “Antimicrobial Resistance” or “Antibiotic Resistance” or AMU” or “Antimicrobial Usage” or “DRI” or “Drug Resistance Infection” or “One Health” or “Antimicrobial Stewardship” or “AMR surveillance” or “GLASS” or “Disease Surveillance” or “Antibiotics”) OR (“IHR” or “International Health Regulations”)]. Using a filter, titles and abstracts in the 6 official WHO languages were put forward for screening. Gray literature and articles in other languages were excluded from title/abstract screening. We excluded gray literature since much of the peer-reviewed literature was dominated by opinion and narrative studies using gray literature as the source. Screening of titles, abstracts and full texts was completed by at least 2 reviewers from a pool of 4 reviewers (J.B., R.J., D.H., R. Ahmad) independently. Fifty-six articles were included in the final review, with one-third of reviewers from this pool reviewing where there was disagreement.

Risk of bias was assessed using the quality criteria for methods set by the Integrated Quality Criteria for Review of Multiple Study Designs (ICROMS) given the heterogeneity of study designs, with assessments presented in Supplementary File 03.^30^

### Statistical Analysis

To ensure rigor in the qualitative thematic analysis, deductive and inductive analyses were conducted. For each approach, the final pool of 56 articles was independently analyzed by 3 coders (J.B., R.J., D.H.) using NVivo software version 14.^31^ The articles were independently coded by 3 researchers, and a fourth reviewer (R. Ahmad) validated the coding quality. A comprehensive codebook was developed to define each code and outline application rules, ensuring consistent and reliable coding across all coders. A tree map was generated in NVivo to compare coding frequencies across the articles. The 3 coders (J.B., R.J., D.H.) met regularly to review codes and themes, engaging in reflexive discussions with the wider researcher team to identify patterns and resolve conflicts, contradictions, and different interpretations. The findings were subsequently reviewed and validated during SEDRIC (Surveillance and Epidemiology of Drug-resistant Infections Consortium) board meetings, where additional input and feedback were incorporated to strengthen the analysis.

### Deductive and inductive analyses

The deductive analysis used the Organization for Economic Co-operation and Development (OECD) Rethinking Health System Performance Assessment Framework.^32^ Developed since the COVID-19 pandemic, this framework incorporates all domains of previous health systems performance frameworks^33^ and aligns with the threshold of “health systems are overwhelmed” in defining a pandemic. Using the framework as part of a deductive coding structure for a rapid review allowed for systematic analysis of how AMR considerations align with pandemic agreement provisions. The framework's 4 cross-cutting dimensions—equity, efficiency, resilience, and sustainability—correspond directly with the foundational themes of pandemic preparedness and response. The framework of choice is also consistent with the recent WHO restructure, consolidating 10 existing divisions into 4 primary divisions, with AMR repositioned under the Health Systems division.

**Figure 2.**
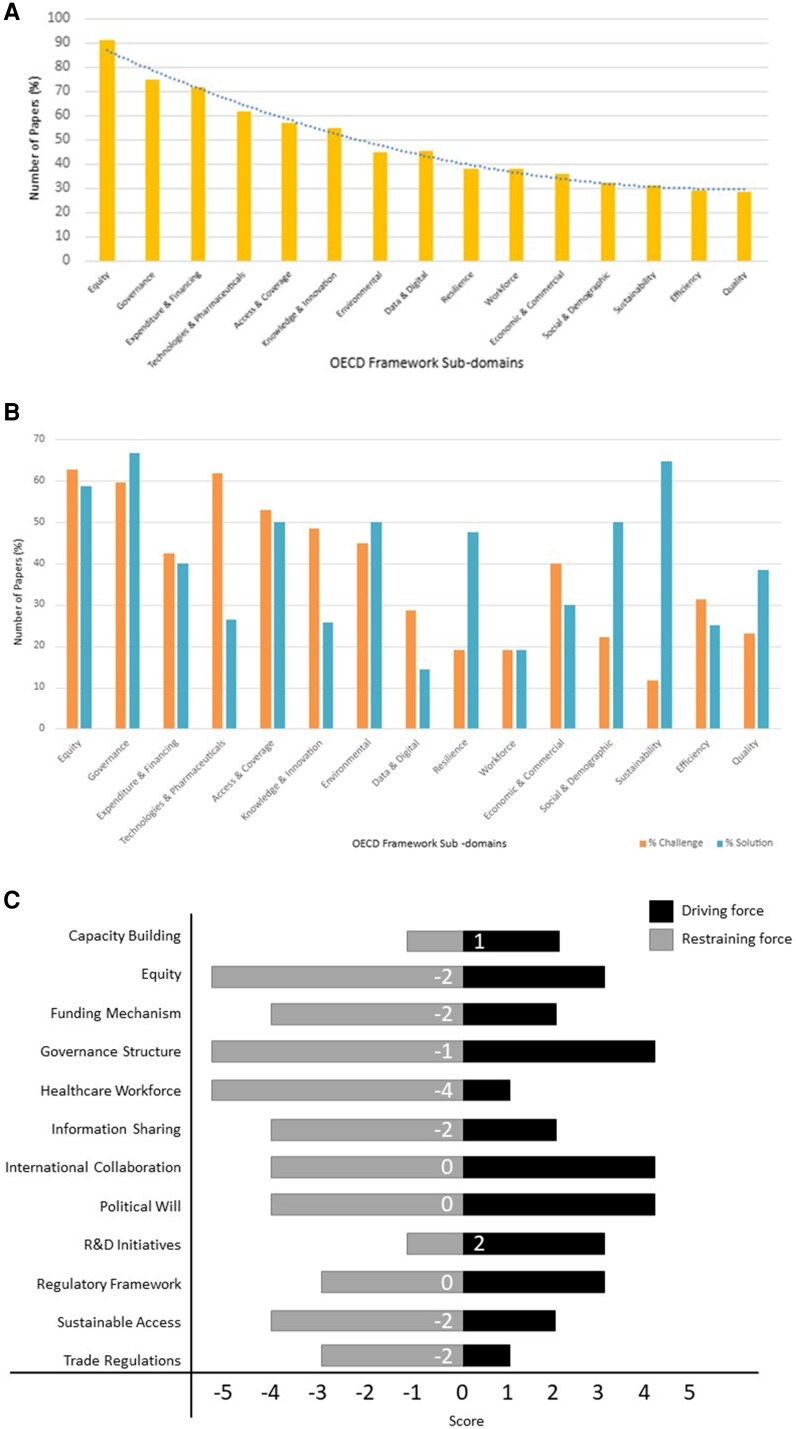
A: Consideration of the subdomains of the OECD health system performance assessment framework. B: Challenges and proposed solutions mapped to the subdomains of the OECD Health System Performance Assessment Framework. C: Key driving and restraining forces for including AMR in the pandemic agreement resulted from the literature and the reviewers' scorings. Abbreviations: AMR, antimicrobial resistance; OECD, Organization for Economic Co-operation and Development; R&D, research and development.

Using line by line coding and content analysis,^34^ the text was mapped to the 15 subdomains within the 4 main domains (Context, Performance, Process & Intervention, Structure) (Supplementary File 02 and Figure 2A).^32^ Following this, the coded text was further categorized into challenges to the implementation of a pandemic agreement and proposed solutions to enable the implementation of a pandemic agreement. The policy domains of each included study were mapped to Kingdon's Multiple Streams Framework,^35^ which conceptualizes policy development across 3 domains: the problem stream, policy stream, and political stream. Mapping studies to these 3 streams enables a structured assessment of where the existing literature concentrates its analytical focus and whether studies indicate convergence across streams that could signal the presence of “policy windows” and opportunities for advancing AMR on the policy agenda (see Table 1).

**Table 1.** Study characteristics.

	Study, year	Corresponding author location	All authors’ location	Aims/research question	Methodology and study period	Policy domain (problem, policy, political)^35^	Framework of analysis
1	Taylor, 2024^36,37^	Colombia	Colombia	Not specified	Expert opinion Study period: not stated	Problem	Not specified
2	Wenham and Stout, 2024^38^	UK	UK	To assess the utilization of PHEIC and pandemic language within national legislation	Scoping review Study period: not stated	Problem	PHEIC
3	Chattu et al, 2024^39^	India; Canada	India; Canada	To explore the role of global health diplomacy (GHD) in pandemic treaty negotiations by providing deep insight into the ongoing drafting process under the WHO leadership	Narrative review Study period: not stated	Problem Policy	Narrative analysis along the domains of politics, policymaking, public health, ethics, management, and others
4	Schwalbe et al, 2024^40^	USA	USA	Not specified	Expert opinion Study period: not stated	Problem	Not specified
5	Wenham and Eccleston-Turner, 2024^41^	UK	UK	Not specified	Expert opinion Study period: not stated	Political	Not specified
6	Mettenleiter and Winkler	Germany	Germany; Norway; USA	Not specified	Expert opinion Study period: not stated	Problem	Not specified
7	Ruckert et al, 2024^42^	Canada	Canada	Suggest that the protocol mechanism of the treaty proposed under Article 31 offers an opportunity to develop a subsidiary agreement (or protocol) to further codify the specific obligations and enforcement mechanisms necessary to meet the treaty's AMR provisions; to highlight experiences with previous treaty implementation that relied on protocols to inform design of a future AMR protocol	Expert opinion Study period: not stated	Policy	Not specified
8	Torreele, 2024^43^	UK	UK	Not specified	Expert opinion Study period: not stated	Problem	Not specified
9	Lehtimaki et al, 2024^44^	USA	Portugal; Malaysia; UK; Palestine; Moldova; USA	Not specified	Expert opinion Study period: not stated	Political	Not specified
10	Mendelson et al, 2024^45^	South Africa; India	South Africa; India; UK; Zambia; Tanzania; USA; Canada; Ethiopia	To provide actionable, evidence-backed solutions to the challenge of AMR and sustainable global antibiotic access	Documentary analysis (qualitative) Study period: not stated	Policy	Analytical commentary
11	Schmidt, 2023^46^	USA	USA	Not specified	Expert opinion Study period: not stated	Problem	Not specified
12	Constantin and Sternstein, 2023^47^	USA	USA	To identify and assess instances of state practice and evidence of *opinio juris* to determine whether a norm of customary international law mandating states to ensure health care workers' freedom of movement during pandemics exist	Documentary analysis (qualitative) Study period: March and April 2020	Problem	Not specified
13	Gostin et al, 2023^48^	USA	USA	Not specified	Expert opinion Study period: not stated	Problem Policy Political	Not specified
14	Kavanagh et al, 2023^49^	USA	UK; USA; Brazil; Kenya	To explore reasons why states comply with international law, even in the absence of sanctions. Drawing on human rights, trade, finance, tobacco, and environmental law, we categorize compliance mechanisms as police patrol, fire alarm, or community organizer models.	Expert opinion Study period: not stated	Problem Policy	Not specified
15	Shakfeh et al, 2023^50^	USA	USA	Not specified	Expert opinion Study period: not stated	Problem Policy	Not specified
16	Khosla et al, 2023^51^	Malaysia	UK; Malaysia	Not specified	Expert opinion Study period: not stated	Problem	Not specified
17	Evaborhene et al, 2023^52^	South Africa	South Africa; Nigeria; UK	To explain how newer mechanisms such as the WHO platform for MCMs could complement ongoing reforms by fostering equitable participation in global health governance	Expert opinion Study period: not stated	Problem Political	Not specified
18	Driece et al, 2023^53^	Thailand	Switzerland; Thailand; South Africa; Netherlands	Not specified	Expert opinion Study period: not stated	Problem	Not specified
19	Gallo-Cajiao et al, 2023^54^	USA	USA; Canada; Austrialia; South Africa	To explore the current institutional landscape for pandemic prevention in light of ongoing negotiations of a so-called pandemic treaty and how prevention of zoonotic spillovers from the wildlife trade for human consumption could be incorporated	Expert opinion Study period: not stated	Problem Policy	Not specified
20	The Lancet Global Health, 2023^55^	UK	UK	Not specified	Expert opinion Study period: not stated	Policy	Not specified
21	Matsoso et al, 2023^56^	Switzerland; Thailand; South Africa; Netherlands	Switzerland; Thailand; South Africa; Netherlands	Not specified	Expert opinion Study period: not stated	Policy	Not specified
22	Phelan, 2023^57^	USA	USA	Not specified	Expert opinion Study period: not stated	Political	Not specified
23	Hanbali et al, 2023^58^	USA	USA; Canada; Malayasia; Palestine	To propose the establishment of an independent monitoring committee to monitor state parties' compliance with and reporting of the pandemic accord	Expert opinion Study period: not stated	Policy	Not specified
24	Hayman and Woolaston, 2023^59^	New Zealand; Australia	New Zealand; Australia	Not specified	Expert opinion Study period: not stated	Policy	Not specified
25	Taylor, 2022^60^	Colombia	Colombia	Not specified	Expert opinion Study period: not stated	Political	Not specified
26	Jackson et al, 2022^61^	Canada	Canada; Australia	To explore that it may be more effective and ethically justifiable for LMICs and civil society to deploy strategic resistance when it comes to activities that may trigger unjustifiable travel restrictions, such as sharing access to pathogens, viral samples, and sequencing data	Expert opinion Study period: not stated	Problem Political	Not specified
27	Hannon et al, 2022^62^	USA; Malaysia	USA; Malaysia	Not specified	Expert opinion Study period: not stated	Problem	Not specified
28	Carlson and Phelan, 2022^63^	USA	USA	To explore how a treaty would provide opportunities to simultaneously expand reporting obligations, accelerate the sharing of scientific discoveries, and strengthen existing legal frameworks, all while addressing the most complex issues that global health governance currently faces	Expert opinion Study period: not stated	Policy	Not specified
29	Wenham et al, 2022^64^	UK; USA	UK; USA	Not specified	Expert opinion Study period: not stated	Problem Policy	Not specified
30	Hodgson et al, 2022^65^	Switzerland	Switzerland	Not specified	Expert opinion Study period: not stated	Political	Not specified
31	Lee and Yeh, 2022^66^	Taiwan	Taiwan	To offer a stronger justification to echo the World Health Assembly (WHA) resolution that stresses the principle of solidarity with all peoples and countries	Expert opinion Study period: not stated	Problem	Not specified
32	Weldon et al, 2022^67,68^	Canada	Canada	To outline the anatomy of the emerging regime complex for AMR. It then considers whether strategies applied in climate governance can be leveraged to improve the coherence of global AMR governance while harnessing the benefits offered by decentralization.	Expert opinion Study period: not stated	Problem Policy	Not specified
33	Lake et al, 2022^69^	Canada	Canada	By illustrating the clear link between the efforts to address both pandemic threats and outlining 6 dual-purpose provisions that could address both pandemic threats, this article makes the case that including AMR in the pandemic instrument makes the most effective use of limited time and resources to ensure the world's best opportunity to prevent, prepare for, and respond to future global pandemics.	Expert opinion Study period: not stated	Problem Policy	Not specified
34	Van Katwyk and Outterson, 2022^70^	USA; Canada	USA; Canada	To explore the inclusion of AMR within the pandemic instrument from 3 perspectives: first, through the lens of global AMR governance; second, from the perspective of technical governance challenges and opportunities affecting the global ability to address AMR and future pandemics; and third, from the perspective of pandemic instrument mechanisms for strengthening global AMR governance	Expert opinion Study period: not stated	Problem	Not specified
35	Van Katwyk et al, 2022^71^	Canada	Canada	To identify key characteristics of an effective unifying global target for AMR based on past experiences of unifying global targets in climate and global health domains	Expert opinion Study period: not stated	Problem Political	Not specified
36	Palkovits et al, 2022^72^	Canada	Canada	As the intergovernmental negotiating body drafts the new pandemic instrument, there is an opportunity to establish smarter global governance arrangements that not only promote but also mandate global intersectoral and interinstitutional equity, cooperation and solidarity, and the One Health perspective vital to the success of pandemic preparedness and response. With this opportunity comes an urgent need to consider the type of mechanism best suited to this purpose. To explore 6 such mechanisms and the possibilities they offer.	Expert opinion Study period: not stated	Problem Policy	Not specified
37	Scott Weese et al, 2022^73^	Canada	Canada; Belgium; Spain; Australia; Italy; France; Switzerland	The objective of this paper is to discuss potential governance approaches to optimizing AMU in animals within a pandemic instrument that uses a broad whole-of-society and whole-of-government One Health approach, while highlighting the inherent and often underappreciated complexities.	Expert opinion Study period: not stated	Problem Political	Not specified
38	Caceres et al, 2022^74^	Canada	Canada; India; Denmark	To examine that the WHO's upcoming international pandemic instrument presents a unique opportunity to support stronger R&D mechanisms for antimicrobials in its framework	Expert opinion Study period: not stated	Problem Policy Political	Not specified
39	Weldon et al, 2022^67,68^	Canada	Canada; Denmark; USA; UK	To explore tools from social science that treaty negotiators can leverage to identify the relevant governance challenges associated with AMR and design a pandemic instrument that incorporates effective solutions to address this urgent threat	Expert opinion Study period: not stated	Problem Policy	Not specified
40	Meier et al, 2022^75^	UK; USA	USA; UK	To examine how the trilogy of reforms fit together, considering: how these reforms can complement each other to support pandemic prevention, preparedness, and response; what financing mechanisms are necessary to ensure sustainable health governance; and why vital norms of equity, social justice, and human rights must underpin this new global health system	Expert opinion Study period: not stated	Problem Policy	Not specified
41	Ren et al, 2022^76^	Canada	Canada; Sweden; USA; India; Ecuador; Zambia	To discuss key components that need to be coordinated and paired with adequate financing and resources to ensure antibiotic effectiveness as a global public good, which should be central while discussing a new global agreement	Expert opinion Study period: not stated	Problem Policy	Not specified
42	Taylor, 2021^77^	Colombia	Colombia	Not specified	Expert opinion Study period: not stated	Problem	Not specified
43	Bauernfeind et al, 2024^78^	Spain	Belgium, Spain, UK, UAE, Israel, Portugal, Iceland, Finland	Not specified	Expert opinion Study period: not stated	Problem Policy	Not specified
44	Taylor, 2025^79^	Colombia	Colombia	Not specified	Expert opinion Study period: not stated	Problem	Not specified
45	Anderson et al, 2025^80^	New Zealand	New Zealand	To evaluate the ethical and public health implications of the revisions to the pandemic treaty, the authors examined changes to the text between the working draft and the proposed agreement.	Comparative analysis (qualitative) Study period: July 13, 2022, and April 22, 2024	Problem Policy	Cosmopolitanism framework
46	Kamin-Friedman et al, 2025^81^	Israel	Israel	To explore the content of the agreed IHR amendments and the “pandemic agreement” draft, detailing the moral and utilitarian rationales for advancing these documents from the Israeli perspective	Expert opinion Study period: 2005 and 2024	Problem Policy Political	Not specified
47	Chen, 2024^82^	China	China	To explore the need to distinguish the functions of the pandemic treaty and the IHR, adopt a soft and hard contracting model, establish an open and transparent pandemic determination mechanism, reform the institutional functions of WHO, and establish an effective dispute settlement mechanism in order to solve the above problems	Expert opinion Study period: December 2021–May 2024	Problem Policy Political	Not specified
48	Lenharo, 2024^83^	USA	USA	Not specified	Expert opinion Study period: not stated	Policy	Not specified
49	Finch et al, 2025^84^	USA	USA; Malaysia; Ghana; Austria; Germany; Uganda; China	To urge WHO member states to adopt the pandemic agreement, with robust measures that champion One Health, pandemic prevention, and global cooperation for health	Expert opinion Study period: not stated	Problem	Not specified
50	Barber, 2024^85^	USA	USA	To offer a counternarrative to claims that WHO is overstepping its historic role in global governance	Expert opinion Study period: not stated	Problem Policy	Not specified
51	Taylor, 2024^36,37^	Colombia	Colombia	Not specified	Expert opinion Study period: not stated	Political	Not specified
52	Renganathan et al, 2025^86^	Italy	Malaysia; Italy; Portugal; Germany; Spain; Brazil; UK	Not specified	Expert opinion Study period: not stated	Policy	Not specified
53	Lee and Piper, 2025^87^	Canada	Canada	Assessed agreed-upon revisions to IHR Article 43 adopted in May 2024 and found no substantive change to the rules and commitments for travel measure use	Expert opinion Study period: not stated	Problem Policy	Not specified
54	Yang et al, 2024^88^	China	China, UK	To clarify China's role in the global antibiotic industry chain by analyzing China's evolving antibiotic trade and exploring the changing trends in the comparative advantage of Chinese-produced antibiotics	Descriptive analysis (quantitative) Study period: 2002 to 2021	Policy	Not specified
55	Ndembi et al, 2024^89,90^	Ethiopia	Ethiopia; Malaysia; Kenya; USA; Morocco; South Africa	Not specified	Expert opinion Study period: not stated	Problem Policy	Not specified
56	Cohen, 2024^91^	USA	USA	Not specified	Expert opinion Study period: not stated	Problem	Not specified

Abbreviations: AMR, antimicrobial resistance; AMU, antimicrobial usage; IHR, International Health Regulations; LMIC, low- and middle-income country; MCM, Medical countermeasures; PHEIC, Public Health Emergency of International Concern; R&D, research and development; WHO, World Health Organization.

### Force-field analysis

A force-field analysis (FFA) was constructed to quantify the identified driving and restraining forces to the proposed pandemic agreement. Force-field analysis is an analytical tool widely used in change management and strategy development.^92^ The analysis with a clear visual to support decision-makers allows consideration of the “net” resultant force. Force-field analysis can be used to help leaders and stakeholders identify, document, and understand forces that are mutable and immutable and plan implementation. However, this process must be carefully executed and transparent to allow for validation and amendments.^92,93^

Key driving forces and restraining forces identified in the inductive analysis were selected by the project team and wider expert panel. Four researchers (R. Ahmad, J.B., R.J., D.H.) then independently scored each factor from ±1 to ±5 (−1 is a weak inhibitor and +5 is a strong facilitator). The mean score across the 4 scores was calculated for each factor and a net force was also included (Figure 2C).

## Results

We identified 242 studies from database searches plus 2 studies from hand searches. After removing 79 duplicates, titles and abstracts of 165 studies were screened, of which 97 studies were eligible for full-text retrieval. Fifty-six studies were included for data extraction and analysis. The PRISMA chart in Figure 1 summarizes this process.

The characteristics of the 56 included studies are presented in Table 1. The majority (90%) were opinion pieces (50); the rest were documentary analyses (2), review articles (2 studies; 1 scoping review and 1 narrative review), and empirical research (2 studies; 1 qualitative and 1 quantitative research). Most studies were published in medical (42%; 21 studies) and interdisciplinary journals (30%; 13 studies). In terms of publication type, these comprised policy analysis (15), commentaries (10), opinion pieces (5), editorials (7), review articles (3), news sections (6), correspondence (3), original research articles (3), essays (2), and 1 study each on personal perspective and analysis. Mapping these studies to Kingdon’s policy domains, the problem stream dominated overall, with 39 of the 56 included studies (70%) focusing critique in this domain and only 4 studies (7%) engaged across all 3 streams.

### Geographical representativeness of authorship

Geographic distribution of where authors are primarily based revealed a concentration of authorship from HICs, particularly in North America and Europe. The United States had the highest representation (*n* = 23),^40,44-50,54,57,58,62-64,67,70,75,76,83-85,89,91,94^ followed by Canada (*n* = 15)^39,42,45,54,61,67-74,76,87^ and the United Kingdom (*n* = 15).^38,41,43-45,49,51,52,55,64,67,75,78,86,88^ Additional contributions included Switzerland (*n* = 4),^53,56,65,73^ Australia (*n* = 4),^54,59,61,73^ China (*n* = 2),^82,88^ Germany (*n* = 3),^84,86,94^ Israel (*n* = 2),^78,81^ Taiwan (*n* = 1),^66^ and New Zealand (*n* = 2).^59,80^ In contrast, contributions from LMICs remain limited, with representation from South Africa (*n* = 6),^45,52-54,56,89^ India (*n* = 4),^39,45,74,76^ Colombia (*n* = 5),^36,37,60,77,79^ Brazil (*n* = 2),^49,86^ Palestine (*n* = 2),^44,58^ Ethiopia (*n* = 2),^45,89^ Kenya (*n* = 2),^49,89^ Ghana (*n* = 1),^84^ Uganda (*n* = 1),^84^ Nigeria (*n* = 1),^52^ and Tanzania (*n* = 1).^45^ Additionally, the corresponding author base, however, was limited to 15 countries: Canada, United States, Netherlands, Switzerland, South Africa, Brazil, Colombia, Thailand, Ethiopia, Italy, Spain, China, New Zealand, Israel, and Malaysia.

### Health system performance domains

Of the 15 subdomains of the OECD framework, the subdomains receiving the most attention in the literature, in order of frequency, are shown in Figure 2A, with Equity (51), Governance (42), and Expenditure & Financing (40) being most prevalent. In contrast, the Workforce subdomain was notably underrepresented, with only 21 articles raising this issue.

### Challenges and solutions to the implementation of a pandemic agreement

Overall, the focus was on challenges to implementation with less focus on solutions. However, 6 subdomains, specifically Governance, Resilience, Social & Demographic, Sustainability, Environment, and Quality, emerged as areas where proposed solutions were considered above challenges identified to implementing a pandemic agreement (Figure 2B, Table 2).

**Table 2.** Text excerpts of subdomains that focused largely on proposed solutions compared with challenges of implementing a pandemic agreement.

	Challenges	Solutions
Governance^39,49,53,54,58,61,63,68,70-73,75,78,80^	The current global health governance structure has failed to provide reasonable and equal access to the effective prevention of pandemics, and the World Health Organization (WHO) has fallen short of its expectations by its delayed responses.^39^	By adopting similar institutional design elements as the Paris Agreement, a pandemic instrument could provide much needed leadership on these specific issues in need of centralization while also being adequately flexible to encompass and permit location-specific initiatives tailored to different national and local circumstances.^68^
	A new top-down global framework for AMR, on the other hand, will likely suffer from the same equity and representational challenges that plague current global health governance structures.^68^	Governance functions and core programmatic activities, such as risk understanding and risk assessment, could be financed through a mix of assessed and voluntary contributions from member countries.^54^
	AMR represents a key global governance challenge that requires equitable global coordination. Existing governance mechanisms, including the International Health Regulations (IHR) are limited in their ability to address AMR amidst deep fragmentation, insufficient governance infrastructure, and concerning global health inequities.^42^	Global health governance, global biodiversity governance, global food governance, and global trade governance should be more effectively coordinated if pandemics are to be prevented.^54^
Expenditure & Financing^45,48,54,69,74-77,82,84,86^	The global health financing architecture is deeply fragmented, which can often lead to clear capacity gaps remaining unfilled and barely visible.^49^ While driven by a commitment to strengthen prevention, this proposal has faced resistance from lower-resourced countries citing concerns about overly prescriptive requirements and implementation challenges given competing national priorities and a lack of guaranteed financial assistance.^84^	Available financing at the outbreak of a pandemic, as well as ahead of emergencies to build capacity in the long term, is an important economic incentive for compliance and is recognized in discussions about common but differentiated responsibilities for preparedness.^67^ The translation of global aspirations into national policies requires more than just setting a goal; it also requires countries to have sufficient resources and capacity to implement the actions needed to achieve the goal. This can be particularly challenging in low- and middle-income countries, where a one-size-fits-all solution may be more burdensome than in high-income countries. Failure to recognize this reality may disincentivize countries from participating in a global effort for which the costs may appear to outweigh the benefits.^49^
Resilience^47,53,68,71,76^	—	In the Zero Draft of the pandemic instrument, it was proposed that strengthening the manufacturing capacity of pandemic response products in low-income and middle-income countries, through transfer of technology and know-how, would fill the gap in global production capacity (located largely in high-income countries), which falls short of being able to supply 8 billion people during a global event.^71^ A more equitable world is one that prevents the conditions that give rise to pandemics, is more prepared, and is more able to respond when outbreaks become pandemics.^53^ Drawing on a hybrid approach and design elements from treaties within climate governance, namely, the Cancún and Paris Agreements, a pandemic instrument can leverage the responsiveness, flexibility, adaptability, and resilience of decentralization and the consistency, reliability, and efficiency of centralization.^68^
Social & Demographic^50,57,64,69,75^	Repeating the mistake of over-relying on technical considerations and failing to give due consideration to socioeconomic implications could hinder the accord's ability to deliver member states' ambitions to avoid the major disruptions that led them to embark on negotiating an accord and amendments processes in the first place. Critically, it could also undermine member states' established aim to ensure equity in prevention and response—especially for those countries and regions with lesser international influence, limited surveillance and reporting capacity, and greater socioeconomic vulnerability.^57^	Member states need to establish a definition with balanced and well-calibrated technical and socioeconomic considerations that can provide holistic situational awareness, especially during the early stages of an outbreak, to avoid repeating past mistakes and to ensure that the accord can achieve the ambition needed.^57^
Sustainability^39,45,50,67,69,73-76^	—	For instance, “to establish a sustainable market, and in return for longer term health security as Alakija argues, African countries will initially have to pay more to cover higher local manufacturing costs”.^50^ To safeguard the effectiveness of antimicrobials, a comprehensive pandemic instrument could mandate the development or use of a regulatory framework governing the use of antimicrobials in a sustainable, acceptable, fair, and effective manner.^69^
Quality^42,43,47,48,51,54,78,85,88^	The global health care workforce faces a dynamic scenario where countries with low health care worker ratios often experience a significant drain of their staff to high-income countries, enticed by the promise of better salaries and living standards. This phenomenon, ultimately, amplifies existing disparities and inequities in health care access and quality.^47^	—

Abbreviation: AMR, antimicrobial resistance.

### Force-field analysis

Figure 2C illustrates the balance between restraining (light shade) and driving (dark shade) forces for incorporating AMR into the pandemic agreement. The analysis revealed that most subthemes resulted in a net opposing force. Notably, the health care workforce subtheme shows the highest restraining net force at −4. Equity, funding mechanisms, information sharing, sustainable access, and trade regulations all reflected a dominance of barriers to include AMR. In contrast, several subthemes, including political will, regulatory framework, and international collaboration, demonstrated a net force of zero. Some subthemes, such as R&D (research and development) initiatives and capacity building, showed a more favorable scenario with a net score of +2 and +1, respectively.

## Discussion

This rapid literature review offers a synthesis of the literature and provides insights to implications of the inclusion of AMR in the pandemic agreement. As global health challenges evolve, AMR remains a pressing concern, not only for its significant health implications but also for its potential to exacerbate existing inequities and undermine pandemic preparedness efforts.^90,95^ The findings of this study, framed within the OECD Rethinking Health System Performance Assessment Framework, conclude that AMR and the pandemic agreement intersect because they are both underpinned by key issues related to governance, equity, and financing in global health systems. Including AMR is therefore important, but the inclusion of AMR in the agreement should not weaken the focus on AMR in relation to the wider pandemic responses.

While many of the challenges identified in this study are applicable to the pandemic agreement in its entirety, we focused on the issues that have the greatest relevance and bearing to AMR. Key areas for consideration and potential ways forward when fully articulating and implementing the agreement are discussed.

### Financing

Financial constraints play a critical role in the feasibility of any agreement that includes AMR. The lack of financial viability and sustainability for pharmaceutical companies to invest in research and development for new antibiotics impedes progress.^74,89,96,97^ Addressing these financial barriers requires innovative financing models and much more is needed than the existing pilots. Schemes such as the delinked subscription model, pandemic fund, the Pan American Health Organization (PAHO) revolving fund,^98^ public–private partnerships, and other tangible mechanisms, which support AMR management, must be rapidly reviewed and developed as options within the agreement, noting risks of crowd-out and fragmentation.^69^ While there has yet been little to no focus on financing mechanisms for diagnostics in AMR, options include pooled procurement, public–private partnerships (eg, via AMR-focused donor initiatives or tiered pricing), and diagnostics-use-conditional reimbursement mechanisms.^97^

### Workforce

Resource limitations contribute and exacerbate workforce deficits,^39,47^ affecting all parts of the care pathway during “peace time.” Suboptimal workforce numbers and high turnover of staff limit the opportunities for training in infection, prevention and control (IPC) and antimicrobial stewardship (AMS) and often have lower priority.^69^ While shortage hampers health systems' efforts to control AMR, AMR also imposes a threat to health care workers. Health care professionals experience a high risk of acquiring AMR infections due to prolonged exposure in health care settings.^99^ Prioritizing the vaccination of health care staff for the recommended schedule of vaccinations and newly available vaccines in the event of a pandemic is crucial to prevent staff absences and protect care pathways. At the same time, we need to understand the determinants to optimal vaccine uptake in health professionals.^24^

Vaccine hesitancy has major social, cultural, and economic implications, potentially eroding public confidence as well as jeopardizing health care provision. Vaccines are critical for addressing AMR by reducing the incidence of infectious diseases and subsequent antibiotic use.^43,100^ Assessing the impact of vaccines on infection by resistant pathogens is a WHO priority^101^ and UNGA recommendation.^3^

Research-based evidence on workforce models receives relatively low levels of funding given the critical need for learning as part of pandemic preparedness and response in the event of a pandemic.^102^ Innovative workforce configurations that help optimize infection prevention and control, and improve access to, and stewardship of effective drugs that are also cost-efficient remain to be established. To ensure effective pandemic preparedness, workforce resilience must be prioritized through systemic, evidence-informed solutions.

### Governance

Lack of effective governance is reflected by weak regulatory frameworks for professions, industry, and licensing; inconsistent policy enforcement; and fragmented international cooperation, which hinder coordinated efforts to tackle AMR.^42,103^ The necessity for robust global health diplomacy and improved trade regulations emerges as a significant theme, reflecting the need for comprehensive and cohesive policies.

When assessing utility of the IHR during the pandemic, Kavanagh and colleagues^49^ highlight 3 major shortcomings—namely, data sharing, multilateral collaboration, and inequities—in vaccine distribution. The current absence of a robust compliance mechanism within the WHO leaves gaps in compliance and enforcement, requiring delineating roles across WHO, the Quadripartite, and national AMR councils. To address these shortcomings, Kavanagh et al^49^ proposed a more robust compliance structure for the IHR (2005),^11^ incorporating independent rapporteurs, civil society reporting, state accountability measures, trust-building efforts, and a platform for resource and assistance requests. These collective, proactive, and transparent measures are yet to be realized, and have the potential for top-down accountability approaches toward global responsibility, which is needed to address AMR.^49,104^

### Equity issues in global health systems

Cross-cutting across all key areas described previously are the innate systemic inequities whereby wealthier nations hold power through ownership of resources, including medical and technological innovations,^63^ with direct implications for addressing AMR. Gaps in regional manufacturing capabilities^43^ result in inadequate access to diagnostics and treatment and essential medical products for infectious diseases for a majority of the population in low-resourced settings.^67^ Between- and within-country inequities exacerbate the impact on the most marginalized groups with lack of access to preventative and curative care for infections.^63,95^ Inequities transcend other areas of pandemic response; during the COVID-19 pandemic, South African researchers shared genomic sequence data for the Omicron variant but with consequences for its transparency, such as travel bans.^90^ While vaccines were trialed in South Africa, availability in this region remained largely inaccessible.

Rapid sharing of the incidence and prevalence of resistant pathogens and their genetic sequence, along with equitable and timely access to diagnostics, is critical. The PABS system represents an essential component of the pandemic agreement, offering a mechanism toward global safety and equity.^90^ While its provisions are legally binding for manufacturers entering into agreements with the WHO, they do not impose binding obligations on participating states. Implications for AMR could include ensuring equitable and affordable access to new antibiotics and rapid diagnostics in LMICs, concrete benefit-sharing, and access to provisions relevant to varying LMIC financing pathways. Medium- and long-term capacity strengthening must include facilitating technology transfer to expand manufacturing capacity, enhancing participation in global AMR research, and strengthening global surveillance for resistant pathogens.

### Limitations

While we used a robust methodology, rapid reviews often rely on a limited number of databases.^59^ Narrower search strategies may omit relevant studies. As the source literature for the analysis includes opinion pieces, there is an inherent bias to highlighting “what is left to be done” rather than examples of success. The public voice was largely missing in the included pool. The skew towards high-income authorship is also noted. The need for empirical work going forward is critical for better informed decision-making.

## Conclusion

While AMR is included in the pandemic agreement, assessing the merits and risks associated with doing so are important to inform the detail and implementation strategy of the agreement itself. The lack of empirical data and analysis to substantiate positions highlights the need for monitoring and evaluation going forward. The “how” to address barriers requires joint learning from interventions or actions for addressing problems of similar scope/problem type, such as climate change.^105^

Strengthening governance frameworks, fostering equity, and ensuring fair access to health resources are imperatives and there is consensus on the criticality of these dimensions. Ensuring robust compliance mechanisms within the IHR is 1 mechanism for holding member states accountable and to facilitate global coordination in both AMR management and pandemic preparedness, but these must be acceptable across geographies and economies in order to be effective.

## Supplementary Material

qxag044_Supplementary_Data

## References

[1] Naghavi M, Vollset SE, Ikuta KS, et al Global burden of bacterial antimicrobial resistance 1990–2021: a systematic analysis with forecasts to 2050. Lancet. 2024;404(10459):1199–1226. 10.1016/S0140-6736(24)01867-1 39299261 PMC11718157

[2] Jonas OB, Irwin A, Berthe FCJ, Le GFG, Marquez PV. Drug-resistant infections: a threat to our economic future. 2017. Accessed September 30, 2024. https://documents1.worldbank.org/curated/en/323311493396993758/pdf/final-report.pdf

[3] United Nations General Assembly (UNGA) . Interactive multi-stakeholder hearing as part of the preparatory process for the 2024 high-level meeting on antimicrobial resistance. 2024. https://www.un.org/pga/78/multi-stakeholder-hearing-on-antimicrobial-resistance/.

[4] United Nations General Assembly (UNGA) . Political declaration of the high-level meeting on antimicrobial resistance. 2024. Accessed September 28, 2024. https://uniatf.who.int/publications/m/item/2024-political-declaration-of-the-high-level-meeting-on-antimicrobial-resistance.

[5] World Health Organization (WHO) . Monitoring and evaluation of the global action plan on antimicrobial resistance: framework and recommended indicators. Published online 2019. Accessed August 13, 2025. https://iris.who.int/bitstream/handle/10665/325006/9789241515665-eng.pdf?sequence=1

[6] Ahmad R, Zhu N, Jain R, et al Systems Policy Analysis for Antimicrobial Resistance Targeted Action (SPAARTA): a research protocol. Wellcome Open Res. 2024;9:700. 10.12688/wellcomeopenres.22923.1 40786641 PMC12332476

[7] Albouy P . Quadripartite Call to Action for One Health for a Safer World. WHO; 2023.

[8] Singh S, Bartos M, Abdalla S, et al Resetting international systems for pandemic preparedness and response. BMJ. 2021;375:e067518. 10.1136/bmj-2021-067518 34840131 PMC8624054

[9] Banerjee A, Coulter A, Goenka S, Hollis A, Majeed A. Research across multiple disciplines to respond to health shocks. BMJ. 2024;387:e078445. 10.1136/bmj-2023-078445 39374960 PMC11450974

[10] Lal A, Erondu NA, Heymann DL, Gitahi G, Yates R. Fragmented health systems in COVID-19: rectifying the misalignment between global health security and universal health coverage. Lancet. 2021;397(10268):61–67. 10.1016/S0140-6736(20)32228-5 33275906 PMC7834479

[11] World Health Organization (WHO) . International health regulations. 2005. Accessed September 3, 2025. https://www.who.int/publications/i/item/9789241580496

[12] World Health Organization (WHO) . Report of the review committee regarding amendments to the International Health Regulations (2005). 2023. Accessed September 3, 2025. https://apps.who.int/gb/wgihr/pdf_files/wgihr2/A_WGIHR2_5-en.pdf

[13] Wilder-Smith A, Osman S. Public health emergencies of international concern: a historic overview. J Travel Med. 2020;27(8):taaa227. 10.1093/jtm/taaa227 33284964 PMC7798963

[14] World Health Organization (WHO) . Pandemic prevention, preparedness and response accord. June 10, 2024. Accessed September 28, 2024. https://www.who.int/news-room/questions-and-answers/item/pandemic-prevention--preparedness-and-response-accord.

[15] Osei J . Intergovernmental Negotiating Body. WHO. 2021.

[16] Cullinan K . Pandemic Treaty Offers Opportunity to Repair Fault Lines in COVID-19 Response—and Address Equity. Health Policy Watch; 2021.

[17] Black C . WHO Member States Agree to Develop Zero Draft of Legally Binding Pandemic Accord in Early 2023. WHO; 2022.

[18] World Health Organization (WHO) . Resumed Ninth Meeting of the Intergovernmental Negotiating Body to Draft and Negotiate a WHO Convention, Agreement or Other International Instrument on Pandemic Prevention, Preparedness and Response. A/INB/9/3 Rev.1 Provisional Agenda Item 2 22 Proposal for the WHO Pandemic Agreement. 2024. Accessed September 28, 2024. https://www.who.int/news-room/events/detail/2024/05/20/default-calendar/ninth-meeting-of-the-intergovernmental-negotiating-body-(inb)-for-a-who-instrument-on-pandemic-prevention-preparedness-and-response-resumed-session-2.

[19] Cullinan K . No pandemic agreement by December as negotiators need ‘more time.’ Health Policy Watch. November 11, 2024. Accessed November 27, 2024. https://healthpolicy-watch.news/no-pandemic-agreement-by-december-as-negotiators-need-more-time/

[20] Cullinan K . Pandemic agreement makes progress but still plenty of sticky details to address. Health Policy Watch. November 15, 2024. Accessed November 27, 2024. https://healthpolicy-watch.news/pandemic-agreement-makes-progress-but-still-plenty-of-sticky-details/

[21] World Health Organization (WHO) . Intergovernmental negotiating body to draft and negotiate a WHO convention, agreement or other international instrument on pandemic prevention, preparedness and response. 2025. Accessed September 24, 2025. https://apps.who.int/gb/ebwha/pdf_files/WHA78/A78_10-en.pdf

[22] WHO Media Team . World Health Assembly agreement reached on wide-ranging, decisive package of amendments to improve the International Health Regulations. WHO. June 1, 2024. Accessed November 27, 2024. https://www.who.int/news/item/01-06-2024-world-health-assembly-agreement-reached-on-wide-ranging--decisive-package-of-amendments-to-improve-the-international-health-regulations--and-sets-date-for-finalizing-negotiations-on-a-proposed-pandemic-agreement.

[23] The Lancet . The pandemic treaty: shameful and unjust. Lancet. 2024;403(10429):781. 10.1016/S0140-6736(24)00410-0 38431338

[24] Cocker D, Fitzgerald R, Brown CS, Holmes A. Protecting healthcare and patient pathways from infection and antimicrobial resistance. BMJ. 2024;387:e077927. 10.1136/bmj-2023-077927 39374953 PMC11450933

[25] US Department of Health and Human Services . COVID-19: U.S. impact on antimicrobial resistance. 2024. Accessed September 28, 2024. https://stacks.cdc.gov/view/cdc/119025/cdc_119025_DS1.pdf.

[26] World Health Organization (WHO) . Global Health Repository. 2022. Accessed June 12, 2025. https://www.who.int/data/gho/data/indicators/indicator-details/GHO/sdg-3-8-1-primary-data-availability-for-uhc-service-coverage-index

[27] Walia K, Mendelson M, Kang G, et al How can lessons from the COVID-19 pandemic enhance antimicrobial resistance surveillance and stewardship? Lancet Infect Dis. 2023;23(8):e301–e309. 10.1016/S1473-3099(23)00124-X 37290476

[28] Smela B, Toumi M, Świerk K, et al Rapid literature review: definition and methodology. J Mark Access Health Policy. 2023;11(1):2241234. 10.1080/20016689.2023.2241234 37533549 PMC10392303

[29] Tricco AC, Lillie E, Zarin W, et al PRISMA extension for scoping reviews (PRISMA-ScR): checklist and explanation. Ann Intern Med. 2018;169(7):467–473. 10.7326/M18-0850 30178033

[30] Zingg W, Castro-Sanchez E, Secci FV, et al Innovative tools for quality assessment: Integrated Quality Criteria for Review of Multiple Study Designs (ICROMS). Public Health. 2016;133:19–37. 10.1016/j.puhe.2015.10.012 26704633

[31] Lumivero . NVivo (version 14). www.lumivero.com. [Computer software]. 2023. https://lumivero.com/products/nvivo/.

[32] Organization for Economic Co-operation and Development (OECD) . Rethinking Health System Performance Assessment. OECD; 2024. 10.1787/107182c8-en

[33] Murray CJ, Frenk J. A framework for assessing the performance of health systems. Bull World Health Organ. 2000;78(6):717–731. 10916909 PMC2560787

[34] Hsieh HF, Shannon SE. Three approaches to qualitative content analysis. Qual Health Res. 2005;15(9):1277–1288. 10.1177/1049732305276687 16204405

[35] Béland D, Howlett M. The role and impact of the multiple-streams approach in comparative policy analysis. J Comp Policy Anal Res Pract. 2016;18(3):221–227. 10.1080/13876988.2016.1174410

[36] Taylor L . Pandemic treaty: panel calls for “reset” to talks as negotiators miss deadline. BMJ. 2024;385:q1193. 10.1136/bmj.q1193 38816014

[37] Taylor L . WHO member states agree better ways to detect health threats and set new deadline for pandemic treaty. BMJ. 2024;385:q1227. 10.1136/bmj.q1227 38834196

[38] Wenham C, Stout L. A legal mapping of 48 WHO member states' inclusion of public health emergency of international concern, pandemic, and health emergency terminology within national emergency legislation in responding to health emergencies. Lancet. 2024;403(10435):1504–1512. 10.1016/S0140-6736(24)00156-9 38527480

[39] Chattu VK, Mol R, Singh B, Reddy KS, Hatefi A. Pandemic treaty as an instrument to strengthen global health security: global health diplomacy at its crux. Health Promot Perspect. 2024;14(1):9–18. 10.34172/hpp.42744 38623344 PMC11016140

[40] Schwalbe N, Hannon E, Lehtimaki S. The new pandemic treaty: are we in safer hands? Probably not. BMJ. 2024;384:q477. 10.1136/bmj.q477 38387977

[41] Wenham C, Eccleston-Turner M. Will the pandemic treaty make it over the line? BMJ. 2024;384:q395. 10.1136/bmj.q395 38359920

[42] Ruckert A, Lake S, Van Katwyk SR. Developing a protocol on antimicrobial resistance through WHO's pandemic treaty will protect lives in future pandemics. Global Health. 2024;20(1):10. 10.1186/s12992-024-01015-1 38297334 PMC10829236

[43] Torreele E . Tackling vaccine inequity in 2023: have we made progress? Expert Rev Vaccines. 2024;23(1):1–4. 10.1080/14760584.2023.2292771 38078804

[44] Lehtimaki S, Hannon E, Hanbali L, et al Where there is a will, there is a way: independent assessment of member state compliance with the pandemic agreement. Lancet Glob Health. 2024;12(1):e18–e19. 10.1016/S2214-109X(23)00515-6 37984382

[45] Mendelson M, Lewnard JA, Sharland M, et al Ensuring progress on sustainable access to effective antibiotics at the 2024 UN General Assembly: a target-based approach. Lancet. 2024;403(10443):2551–2564. 10.1016/S0140-6736(24)01019-5 38797179

[46] Schmidt H . Equity needs to be (even) more central under the WHO pandemic agreement. J Med Ethics. 2023;49(12):797–798. 10.1136/jme-2023-109720 37996111

[47] Constantin A, Sternstein A. Healthcare workers' freedom of movement in times of pandemics: an emerging norm of customary international law. Global Health. 2023;19(1):83. 10.1186/s12992-023-00985-y 37950291 PMC10636805

[48] Gostin LO, Klock KA, Finch A. Making the world safer and fairer in pandemics. Hastings Cent Rep. 2023;53(6):3–10. 10.1002/hast.1538 38131499

[49] Kavanagh MM, Wenham C, Massard da Fonseca E, et al Increasing compliance with international pandemic law: international relations and new global health agreements. Lancet. 2023;402(10407):1097–1106. 10.1016/S0140-6736(23)01527-1 37678291

[50] Shakfeh N, Rahman FA, Bertram K. We need a universally endorsed definition of a pandemic for the Pandemic Accord to be effective. BMJ. 2023;382:1946. 10.1136/bmj.p1946 37611960

[51] Khosla R, McCoy D, Marriot A. Backsliding on human rights and equity in the Pandemic Accord. Lancet. 2023;401(10393):2019–2021. 10.1016/S0140-6736(23)01118-2 37271154

[52] Evaborhene NA, Oga JO, Nneli OV, Mburu S. The WHO pandemic treaty: where are we on our scepticism? BMJ Glob Health. 2023;8(6):e012636. 10.1136/bmjgh-2023-012636 PMC10276959 37316254

[53] Driece RA, Matsoso P, da Silva Nunes T, Soliman A, Taguchi K, Tangcharoensathien V. A WHO pandemic instrument: substantive provisions required to address global shortcomings. Lancet. 2023;401(10386):1407–1410. 10.1016/S0140-6736(23)00687-6 37028440 PMC10072861

[54] Gallo-Cajiao E, Lieberman S, Dolšak N, et al Global governance for pandemic prevention and the wildlife trade. Lancet Planet Health. 2023;7(4):e336–e345. 10.1016/S2542-5196(23)00029-3 37019574 PMC10069821

[55] The Lancet Global Health . WHO's pandemic treaty: promises of equity should be kept. Lancet Glob Health. 2023;11(4):e475. 10.1016/S2214-109X(23)00121-3 36870357

[56] Matsoso P, Driece R, Da Silva Nunes T, Soliman A, Taguchi K, Tangcharoensathien V. Negotiating a pandemic accord: a promising start. BMJ. 2023;380:506. 10.1136/bmj.p506 36863729

[57] Phelan AL . The World Health Organization's pandemic treaty. BMJ. 2023;380:463. 10.1136/bmj.p463 36854465

[58] Hanbali L, Lehtimaki S, Hannon E, McNab C, Schwalbe N. Independent monitoring for the pandemic accord: a non-negotiable provision. Lancet. 2023;401(10376):553. 10.1016/S0140-6736(23)00126-5 36736333

[59] Hayman DTS, Woolaston K. Pandemic treaty—incorporate a One Health framework. Nature. 2023;613:27. 10.1038/d41586-022-04565-9 36596952

[60] Taylor L . WHO member states agree to draft international pandemic accord. BMJ. 2022;379:o2975. 10.1136/bmj.o2975 36593564

[61] Jackson C, Habibi R, Forman L, Silva DS, Smith MJ. Between rules and resistance: moving public health emergency responses beyond fear, racism and greed. BMJ Glob Health. 2022;7(12):e009945. 10.1136/bmjgh-2022-009945 PMC9723907 36593643

[62] Hannon E, Hanbali L, Lehtimaki S, Schwalbe N. Why we still need a pandemic treaty. Lancet Glob Health. 2022;10(9):e1232–e1233. 10.1016/S2214-109X(22)00278-9 35841922 PMC9278883

[63] Carlson CJ, Phelan AL. International law reform for One Health notifications. Lancet. 2022;400(10350):462–468. 10.1016/S0140-6736(22)00942-4 35810748

[64] Wenham C, Reisdorf R, Asthana S. Pandemic treaty: a chance to level up on equity. BMJ. 2022;377:o1279. 10.1136/bmj.o1279 35595286

[65] Hodgson TF, Carmona MS, Podmore M. States cannot negotiate a pandemic treaty alone. BMJ. 2022;377:o1281. 10.1136/bmj.o1281 35595277

[66] Lee PH, Yeh MJ. From security to solidarity: the normative foundation of a global pandemic treaty. J Glob Health. 2022;12:03025. 10.7189/jogh.12.03025 35567591 PMC9107287

[67] Weldon I, Liddell K, Van Katwyk SR, et al A pandemic instrument can start turning collective problems into collective solutions by governing the common-pool resource of antimicrobial effectiveness. J Law Med Ethics. 2022;50(S2):17–25. 10.1017/jme.2022.75 36889344 PMC10009384

[68] Weldon I, Yaseen S, Hoffman SJ. A pandemic instrument can optimize the regime complex for AMR by striking a balance between centralization and decentralization. J Law Med Ethics. 2022;50(S2):26–33. 10.1017/jme.2022.76 36889353 PMC10009380

[69] Lake SJ, Van Katwyk SR, Hoffman SJ. Antimicrobial resistance must be included in the pandemic instrument to ensure future global pandemic readiness. J Law Med Ethics. 2022;50(S2):9–16. 10.1017/jme.2022.74 36889351

[70] Van Katwyk SR, Outterson K. Introduction: AMR belongs in the pandemic instrument. J Law Med Ethics. 2022;50(S2):6–8. 10.1017/jme.2022.73 36889349

[71] Van Katwyk SR, Wilson L, Weldon I, Hoffman SJ, Poirier MJP. Adopting a global AMR target within the pandemic instrument will act as a catalyst for action. J Law Med Ethics. 2022;50(S2):64–70. 10.1017/jme.2022.101 PMC10009368 36889348

[72] Palkovits M, Van Katwyk SR, Hoffman SJ. Embed multisectoral governance mechanisms in the pandemic instrument for One Health action. J Law Med Ethics. 2022;50(S2):71–81. 10.1017/jme.2023.30 36889347 PMC10009389

[73] Scott Weese J, Da Costa Junior GA, Gonzalez-Zorn B, et al Governance processes and challenges for reservation of antimicrobials exclusively for human use and restriction of antimicrobial use in animals. J Law Med Ethics. 2022;50(S2):55–63. 10.1017/jme.2022.80 PMC10009376 36889346

[74] Caceres AM, Singh KK, Minssen T, Van Katwyk SR, Hoffman SJ. Using the international pandemic instrument to revitalize the innovation ecosystem for antimicrobial R&D. J Law Med Ethics. 2022;50(S2):47–54. 10.1017/jme.2022.79 PMC10009383 36889345

[75] Meier BM, Habibi R, Gostin LO. A global health law trilogy: transformational reforms to strengthen pandemic prevention, preparedness, and response. J Law Med Ethics. 2022;50(3):625–627. 10.1017/jme.2022.103 36398645

[76] Ren M, So AD, Chandy SJ, et al Equitable access to antibiotics: a core element and shared global responsibility for pandemic preparedness and response. J Law Med Ethics. 2022;50(S2):34–39. 10.1017/jme.2022.77 36889350 PMC10009365

[77] Taylor L . World Health Organization to begin negotiating international pandemic treaty. BMJ. 2021;375:n2991. 10.1136/bmj.n2991 34857541

[78] Bauernfeind A, Reid J, Mccallum A, et al No time to lose: pandemic agreement—urgency over complacency; unity over fragmentation. Int J Health Plann Manage. 2024;39(6):1810–1818. 10.1002/hpm.3847 39133762

[79] Taylor L . Pandemic accord: global health game changer or empty promises? BMJ. 2025;389:r787. 10.1136/bmj.r787 40262834

[80] Anderson EMR, Fenton E, Crump JA. Pandemic treaty textual analysis: ethics and public health implications. J Public Health (Bangkok). 2025;47:837–846. 10.1093/pubmed/fdaf040 PMC12669987 40237606

[81] Kamin-Friedman S, Davidovitch N, Levine H, Nitzan D. IHR amendments and the “pandemic agreement”: an Israeli perspective. Isr J Health Policy Res. 2025;14(1):13. 10.1186/s13584-025-00676-6 40083009 PMC11905496

[82] Chen W . Study on major legal issues and solutions in pandemic treaty negotiations. Front Public Health. 2024;12:1413036. 10.3389/fpubh.2024.1413036 39371209 PMC11449756

[83] Lenharo M . Hope for global pandemic treaty rises—despite missed deadline. Nature. 2024;630(8016):282–282. 10.1038/d41586-024-01658-5 38831085

[84] Finch A, Vora NM, Hassan L, et al The promise and compromise of the WHO pandemic agreement for spillover prevention and One Health. Lancet. 2025;405(10492):1800–1802. 10.1016/S0140-6736(25)00632-4 40188839

[85] Barber M . Technology transfer, intellectual property, and the fight for the soul of WHO. PLOS Glob Public Health. 2024;4(12):e0003940. 10.1371/journal.pgph.0003940 39637131 PMC11620680

[86] Renganathan E, Tediosi F, Abecasis A, et al Balancing equity and global health security towards a fair and effective pandemic agreement. Int J Public Health. 2025;70:1608581. 10.3389/ijph.2025.1608581 40313790 PMC12043451

[87] Lee K, Piper J. Latest revisions to the International Health Regulations will fail to prevent future travel chaos. BMJ Glob Health. 2025;10(1):e017077. 10.1136/bmjgh-2024-017077 PMC11781136 39880415

[88] Yang Y, Husain L, Huang Y. China's position and competitiveness in the global antibiotic value chain: implications for global health. Global Health. 2024;20(1):87. 10.1186/s12992-024-01089-x 39695773 PMC11656612

[89] Ndembi N, Dereje N, Nonvignon J, et al Financing pandemic prevention, preparedness and response: lessons learned and perspectives for future. Global Health. 2024;20(1):65. 10.1186/s12992-024-01066-4 39169389 PMC11337782

[90] Ndembi N, Dereje N, Rahman FA, et al The pandemic agreement: achieving an African win for health security inequity. J Public Health Afr. 2024;15(1):618. 10.4102/jphia.v15i1.618 39145291 PMC11321139

[91] Cohen J . Negotiations on global plan to fight pandemics end without a deal. AAAS. 2024. Accessed September 28, 2024. 10.1126/science.z4n4bvd.

[92] Shafaghat T, Zarchi MKR, Nasab MHI, Kavosi Z, Bahrami MA, Bastani P. Force field analysis of driving and restraining factors affecting the evidence-based decision-making in health systems; comparing two approaches. J Educ Health Promot. 2021;10(1):419. 10.4103/jehp.jehp_1142_20 35071625 PMC8719555

[93] Arab-Zozani M, Zakaria Pezeshki M, Khodayari- Zarnaq R, Janati A. Balancing overuse and underuse in the Iranian healthcare system: a force field theory analysis. Ethiop J Health Sci. 1970;29(2):231–238. 10.4314/ejhs.v29i2.10 PMC6460443 31011271

[94] Alders R, Amuasi JH, Ashraf S, et al Draft of WHO pandemic agreement plays down primary prevention. Lancet. 2024;403(10426):525–526. 10.1016/S0140-6736(24)00066-7 38341244

[95] Rad J . Health inequities: a persistent global challenge from past to future. Int J Equity Health. 2025;24(1):148. 10.1186/s12939-025-02526-y 40410748 PMC12103002

[96] Chandy SJ . Financing antimicrobial resistance interventions: optimizing current and new funding mechanisms for mitigation. 2024. Accessed September 28, 2025. https://icars-global.org/knowledge/financing-antimicrobial-resistance-interventions-optimising-current-and-new-funding-mechanisms-for-mitigation/.

[97] Fleming Initiative . Towards a global AMR diagnostics consortium: strategies and partnerships. 2025. Accessed September 3, 2025. https://www.fleminginitiative.org/eventlisting/towards-a-global-amr-diagnostics-consortium:-strategies-and-partnerships

[98] Holmer H, Reid-Henry S, Agyepong IA, Songane FF, Heymann DL. Pandemic financing should learn from revolving funds. Lancet. 2025;405(10473):122–123. 10.1016/S0140-6736(24)02633-3 39709980

[99] Cocker D, Birgand G, Zhu N, et al Healthcare as a driver, reservoir and amplifier of antimicrobial resistance: opportunities for interventions. Nat Rev Microbiol. 2024;22:636–649. 10.1038/s41579-024-01076-4 39048837

[100] Adeshina OO, Nyame S, Milner J, Milojevic A, Asante KP. Barriers and facilitators to nationwide implementation of the malaria vaccine in Ghana. Health Policy Plan. 2023;38(1):28–37. 10.1093/heapol/czac077 36083007 PMC9825729

[101] Bertagnolio S, Dobreva Z, Centner CM, et al WHO global research priorities for antimicrobial resistance in human health. Lancet Microbe. 2024;5(11):100902. 10.1016/S2666-5247(24)00134-4 39146948 PMC11543637

[102] Walshe K, Smith J, Lamont T, Leary A. Workforce research priorities for resilience to future health shocks—and the workforce crisis. BMJ. 2024;387:e078997. 10.1136/bmj-2023-078997 39374961 PMC11450878

[103] Patel J, Moghaddam SS, Ranganathan S, et al Global policy responses to antimicrobial resistance, 2021–22: a systematic governance analysis of 161 countries and territories. Lancet Infect Dis. 2026;26(1):55–66. 10.1016/S1473-3099(25)00406-2 40914183

[104] Weldon I, Hoffman SJ. Bridging the commitment-compliance gap in global health politics: lessons from international relations for the global action plan on antimicrobial resistance. Glob Public Health. 2021;16(1):60–74. 10.1080/17441692.2020.1788623 32623966

[105] Harring N, Krockow EM. The social dilemmas of climate change and antibiotic resistance: an analytic comparison and discussion of policy implications. Humanit Soc Sci Commun. 2021;8(1):125. 10.1057/s41599-021-00800-2

