# Supplementary material for: Inclusion of antimicrobial resistance in a pandemic agreement: why it matters and what comes next?
Source: Health Aff Sch. 2026 Feb 28;4(3):qxag044. doi: 10.1093/haschl/qxag044 (PMC12975186; doi:10.1093/haschl/qxag044)
Supplement: qxag044_Supplementary_Data [file qxag044_supplementary_data.zip › Supplementary File 1 Inclusion & Exclusion Criteria.docx]

Supplementary file 01: Inclusion and exclusion criteria

| INCLUSION CRITERIA | EXCLUSION CRITERIA |
| --- | --- |
| Study design: Peer-reviewed articles discussing the inclusion of either AMR or the IHR within the context of the Pandemic agreement/treaty  Search terms:  “pandemic treaty”, “pandemic accord”, “pandemic instrument” “pandemic agreement” AND [(“AMR” or “Antimicrobial Resistance” or “Antibiotic Resistance” or “AMU” or “Antimicrobial Usage” or “DRI” or “Drug Resistance Infection” or “One Health” or “Antimicrobial Stewardship” or “AMR surveillance” or “GLASS” or “Disease Surveillance” or “Antibiotics”) OR (“IHR” or “International Health Regulations”)] | Study design: Grey literature or articles which discuss Vaccines (without mention of AMR) in the Pandemic agreement/treaty  Articles not discussing AMR or the IHR within the context of the pandemic agreement/treaty |
| Setting: All | Setting: No exclusions |
| Country: No restriction - all high-income countries (HICs) and low-income and middle-income countries (LMICs). | Country: No exclusions |
| Language: Six official WHO languages (Arabic, Chinese, English, French, Russian and Spanish) | Language: All other languages. |
| Date range: Studies published between 1 December 2021 and 31 May 2025 | Date range: Studies published before 1 December 2021 or after 31 May 2025 |
