# Supplementary figures and images for: Inclusion of antimicrobial resistance in a pandemic agreement: why it matters and what comes next?
Source: Health Aff Sch. 2026 Feb 28;4(3):qxag044. doi: 10.1093/haschl/qxag044 (PMC12975186; doi:10.1093/haschl/qxag044)

Supplementary file 02: OECD Rethinking Health System Performance Assessment Framework


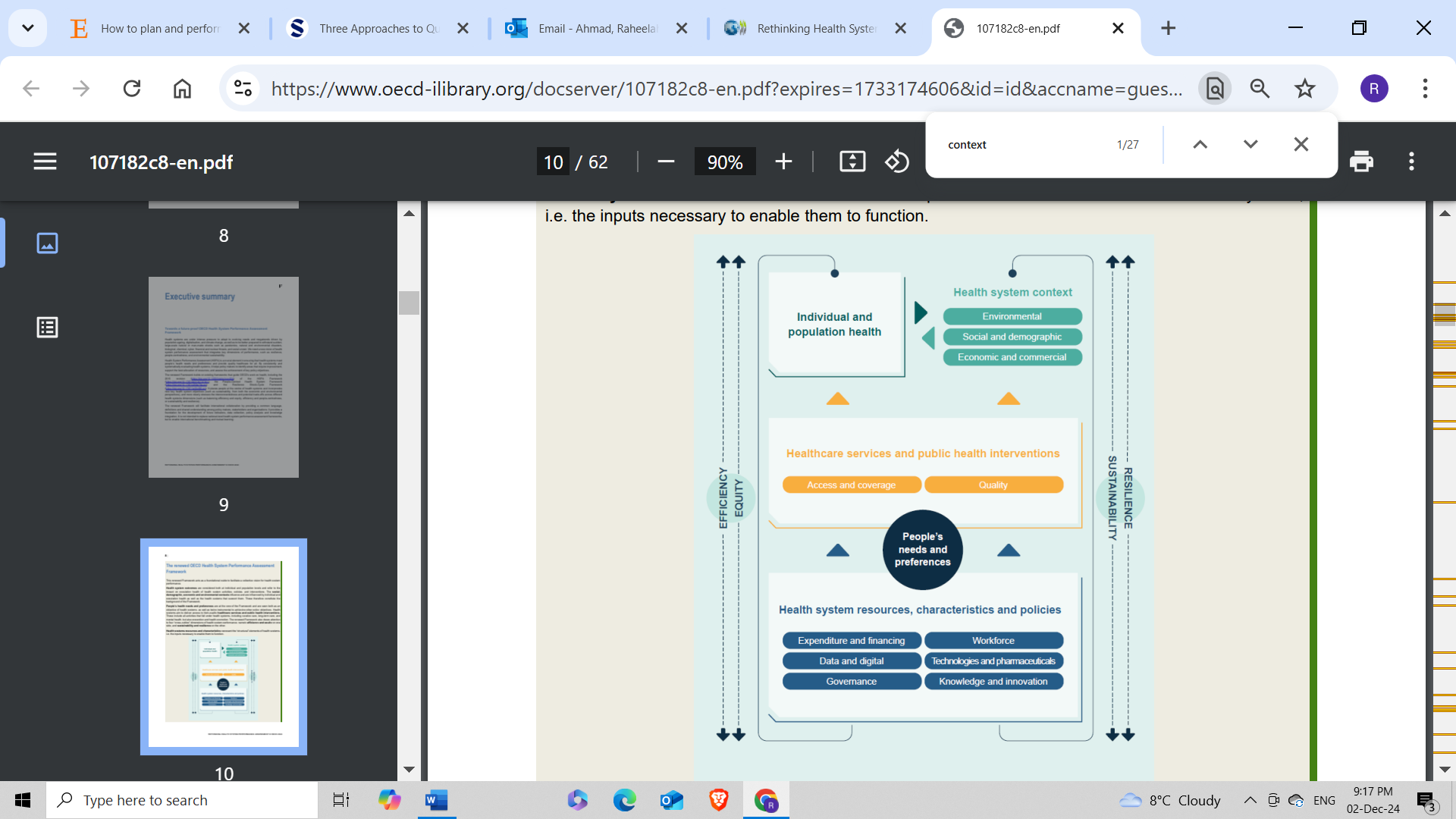

Supplement: qxag044_Supplementary_Data [file qxag044_supplementary_data.zip › Supplementary File 2 OECD Framework.docx]
